# Is fine needle aspiration biopsy reliable in the diagnosis of parotid tumors? Comparison of preoperative and postoperative results and the factors affecting accuracy^☆^

**DOI:** 10.1016/j.bjorl.2018.04.015

**Published:** 2018-06-11

**Authors:** Fazilet Altin, Yalcin Alimoglu, Resit Murat Acikalin, Husamettin Yasar

**Affiliations:** Health Sciences University, Haseki Training and Research Hospital, Otolaryngology Department, Istanbul, Turkey

**Keywords:** Parotid gland, Parotidectomy, Fine-needle aspiration biopsy, Diagnostic accuracy, Glândula parótida, Parotidectomia, Punção aspirativa com agulha fina, Acurácia diagnóstica

## Abstract

**Introduction:**

Fine needle aspiration biopsy is a valuable tool in preoperative evaluation of head and neck tumors. However, its accuracy in management of salivary gland tumors is debatable.

**Objective:**

We aimed to investigate the efficacy and the accuracy of fine needle aspiration biopsy in parotid gland tumors.

**Methods:**

Patients who underwent parotidectomy between January 2008 and June 2017 due to parotid gland tumor were examined retrospectively. Patients with both preoperative fine needle aspiration biopsy and postoperative surgical pathologies were included. Preoperative fine needle aspiration biopsy was categorized as benign, malignant or suspicious for malignancy. Surgical pathology was grouped as benign or malignant. Surgical pathology was compared with fine needle aspiration biopsy, and sensitivity, specificity, accuracy and agreement between both tests were investigated.

**Results:**

217 cases were evaluated and 23 cases were excluded because the fine needle aspiration biopsy diagnosis was non-diagnostic or unavailable. 194 cases were included. The mean age of the patients was 47.5 ± 15.88 (7–82). There were 157 benign, 37 malignant cases in fine needle aspiration biopsy, 165 benign and 29 malignant cases in surgical pathology. The most common benign tumor was pleomorphic adenoma (43.3%), and malignant tumor was mucoepidermoid carcinoma (4.13%). The diagnostic accuracy for fine needle aspiration biopsy when detecting malignancy was 86.52%. Sensitivity and specificity were 68.96% and 89.63% respectively. Positive predictive value was 54.05% and negative predictive value was 94.23%. There was moderate agreement between fine needle aspiration biopsy and surgical pathology (*κ* = 0.52). The sensitivity was 54.54% in tumors less than 2 cm while 77.77% in larger tumors. In tumors extending to the deep lobe, sensitivity was 80%.

**Conclusion:**

Fine needle aspiration biopsy is an important diagnostic tool for evaluating parotid gland tumors. It is more accurate in detecting benign tumors. In tumors greater than 2 cm and extending to the deep lobe, the sensitivity of fine needle aspiration biopsy is high. The use of fine needle aspiration biopsy in conjunction with clinical and radiological evaluation may help to reduce false positive and false negative results.

## Resumo

**Introdução:**

A punção aspirativa com agulha fina é uma ferramenta valiosa na avaliação pré-operatória de tumores de cabeça e pescoço. No entanto, sua precisão no tratamento de tumores de glândulas salivares é discutível.

**Objetivo:**

Nosso objetivo foi investigar a eficácia e precisão da punção aspirativa com agulha fina nos tumores da glândula parótida.

**Método:**

Pacientes submetidos à parotidectomia entre janeiro de 2008 e junho de 2017 por tumor de glândula parótida foram examinados retrospectivamente. Foram incluídos pacientes com punção aspirativa com agulha fina pré-operatória e histopatologia cirúrgica pós-operatória. A punção aspirativa com agulha fina pré-operatória foi categorizada como benigna, maligna ou com suspeita de malignidade. O histopatológico cirúrgico foi agrupado como benigno ou maligno. Os exames histopatológicos foram comparados com a punção aspirativa com agulha fina, e a sensibilidade, especificidade, acurácia e concordância entre os dois testes foram investigadas.

**Resultados:**

Foram avaliados 217 casos e excluídos 23 porque o diagnóstico da punção aspirativa com agulha fina não foi conclusivo ou estava indisponível. Portanto, foram incluídos 194 casos. A média de idade dos pacientes foi de 47,5 ± 15,88 (7–82). Havia 157 casos benignos, 37 malignos na punção aspirativa com agulha fina e 165 casos benignos e 29 malignos na histopatologia. O tumor benigno mais comum foi o adenoma pleomórfico (43,3%) e o tumor maligno mais comum foi o carcinoma mucoepidermoide (4,13%). A acurácia diagnóstica da punção aspirativa com agulha fina na detecção de malignidade foi de 86,52%. A sensibilidade e especificidade foram de 68,96% e 89,63%, respectivamente. O valor preditivo positivo foi de 54,05% e o valor preditivo negativo foi de 94,23%. Houve concordância moderada entre a punção aspirativa com agulha fina e histopatológico (κ = 0,52). A sensibilidade foi 54,54% em tumores maiores do que e 77,77% em tumores maiores. Nos tumores que se estendiam até o lobo profundo, a sensibilidade foi de 80%.

**Conclusão:**

A punção aspirativa com agulha fina é uma importante ferramenta diagnóstica na avaliação dos tumores da glândula parótida. É mais precisa na detecção de tumores benignos. Em tumores maiores do que 2 cm que se estendem até o lobo profundo, a sensibilidade da punção aspirativa com agulha fina é alta. O uso dessa ferramenta em conjunto com a avaliação clínica e radiológica pode ajudar a reduzir os resultados falso-positivos e falso-negativos.

## Introduction

Major salivary gland tumors account for 3% of head and neck cancers.^1^ Benign tumors are more common than malignant tumors. 85% originate from the parotid gland, while submandibular and sublingual gland tumors are less common. Pleomorphic adenoma is most common benign and mucoepidermoid carcinoma is the most common malignant tumor. Some systemic diseases such as metastatic cancers, inflammatory conditions, and lymphoma may also cause parotid gland masses.^1^, ^2^

Fine needle aspiration biopsy (FNAB) is a valuable tool in the preoperative evaluation of head and neck cancers. FNAB for parotid gland lesions has been used for more than 40 years.^2^ It is accepted by most clinicians that it is superior to physical examination and imaging in differential diagnosis of malignant and benign tumors, however some clinicians are insecure about their utility. Preoperative benign and malignant differentiation of parotid gland tumors may be useful both for surgical planning and patient counseling.

We aimed to investigate the diagnostic efficacy and accuracy of FNAB in parotid gland tumors and the factors affecting this in our study.

## Methods

Between January 2008 and June 2017, 217 cases which have undergone parotidectomy due to parotid gland tumors in our department were examined retrospectively. Cases of whom FNAB or final histopathological diagnoses were unavailable or non-diagnostic were excluded. Age, gender, side, extension to the deep lobe, the size of the tumor as measured by ultrasonography, preoperative FNAB diagnosis and final histopathological diagnosis were noted.

FNAB is performed the outpatient setting using 23 gauge needle and 10 cc syringe without local anesthesia. The needle is inserted from a single point and moved in 4–5 directions through the tumor without exiting. After obtaining enough samples, the needle is withdrawn and detached from the syringe. The aspirate is sprayed on at least 3–4 glass slides, smeared, fixed in alcohol and sent to the pathology lab.

Preoperative FNAB diagnosis was classified as benign, suspicious for malignancy or malignant. If possible, the subtypes were noted. Final histopathological diagnosis was grouped as benign and malignant, and typing was noted. The FNAB diagnoses of malignant and suspicious for malignancy and final histopathological diagnosis of malignancy were categorized as positive, and other benign results as negative.

The cases are classified as true negative (FNAB and final histopathological diagnosis are benign), false positive (FNAB diagnosis is malignant, final histopathological diagnosis is benign), true positive (FNAB diagnosis and final histopathological diagnosis are malignant) and false negative (FNAB diagnosis is benign, final histopathological diagnosis is malignant). Sensitivity, specificity, negative predictive value, positive predictive value, accuracy and agreement between both tests were investigated by comparing FNAB and final histopathological diagnosis.

Multinomial logistic regression analysis was performed to investigate any possible effect of age, gender, side, deep lobe involvement, and size according to ultrasonography on true positive, true negative, false positive and false negative results. Cases were grouped according to the parameters found to be significant and then the sensitivity, specificity, negative and positive predictive value and accuracy were investigated for each group (true positive, true negative, false positive, false negative, tumor size and deep lobe extension).

Our study was conducted with the approval of Haseki Training and Research Hospital Ethics Committee (14.09.2017/549). Our study was carried out in concordance with The Code of Ethics of the World Medical Association (Declaration of Helsinki). Informed consent was obtained from all patients participating the study.

## Results

Of the 217 retrospectively investigated patients, 23 were excluded due to unavailable or non-diagnostic FNAB results, 194 cases were examined retrospectively. The age of the patients was 47.5 ± 15.88 (7–82). 88 (45.36%) of patients were female and 106 (54.64%) were male. 100 cases were located in the right parotid gland (51.54%), 94 cases were located in the left parotid gland (48.46%); 166 tumors were in the superficial lobe and deep lobe involvement was present in 28 cases. The size of the tumor was 2.76 ± 1.22 (1.1–9.5) cm.

There were 157 benign, 37 suspicious for malignancy and malignant cases on FNAB, 165 benign and 29 malignant cases in final histopathological diagnosis. The most common benign lesion was pleomorphic adenoma (43.3%); the most common malignant tumor was mucoepidermoid carcinoma (4.13%). True negative, false positive, true positive and false negative cases are shown in Table 1, Table 2, Table 3, Table 4.

**Table 1:** FNAB diagnosis and final histopathological diagnosis of true negative cases.

FNAB diagnosis	Patients	Final histopathological diagnosis	Patients
Pleomorphic adenoma	75	Pleomorphic adenoma	72
		Warthin's tumor	3
Warthin's tumor	38	Warthin's tumor	33
		Oncocytoma	2
		Tuberculosis	2
		Pleomorphic adenoma	1
Lymphoid hyperplasia	6	Lymphadenoma	5
		Chronic sialadenitis	1
Lipoma	4	Lipoma	4
Inflammation	8	Pilomatrixoma	2
		Chronic sialadenitis	2
		Benign epithelial cyst	2
		Chronic granulomatous inflammation	1
		Warthin's tumor	1
Cyst	8	Benign epithelial cyst	7
		Chronic granulomatous inflammation	1
Monomorphic adenoma	2	Basal cell adenoma	2
Benign epithelial tumor	6	Pleomorphic adenoma	3
		Warthin's tumor	3
Total	148	True diagnosis	130
		False diagnosis	18

FNAB, fine needle aspiration biopsy.

**Table 2:** FNAB diagnosis and final histopathological diagnosis of false positive cases.

FNAB diagnosis	Patients	Final histopathological diagnosis	Patients
Suspicious for malignancy	15	Pleomorphic adenoma	6
		Warthin's tumor	5
		Chronic sialadenitis	2
		Myoepithelioma	1
		Monomorphic adenoma	1
Malignant	2		
Acinic cell carcinoma	1	Pleomorphic adenoma	1
Mucoepidermoid carcinoma	1	Warthin's tumor	1
Total	17		

FNAB, fine needle aspiration biopsy.

**Table 3:** FNAB diagnosis and final histopathological diagnosis of true positive cases.

FNAB diagnosis	Patients	Final histopathological diagnosis	Patients
Suspicious for malignancy	14	Mucoepidermoid carcinoma	4
		Acinic cell carcinoma	3
		Adenoid cystic carcinoma	2
		Diffuse B cell lymphoma	2
		Squamous cell carcinoma	1
		Tubular carcinoma	1
		Basal cell carcinoma	1
Malignant	5		
Squamous cell carcinoma	2	Squamous cell carcinoma	2
Adenoid cystic carcinoma	1	Adenoid cystic carcinoma	1
Ductal carcinoma	1	Ductal carcinoma	1
Mucoepidermoid carcinoma	1	Mucoepidermoid carcinoma	1
Total	19		2

FNAB, fine needle aspiration biopsy.

**Table 4:** FNAB diagnosis and final histopathological diagnosis of false negative cases.

FNAB diagnosis	Patients	Final histopathological diagnosis	Patients
Benign	2	Acinic cell carcinoma	1
		Mucoepidermoid carcinoma	1
Warthin's tumor	4	Mucoepidermoid carcinoma	1
		MALT lymphoma	1
		Adenocarcinoma	1
		Diffuse B cell lymphoma	1
Pleomorphic adenoma	3	Mucoepidermoid carcinoma	1
		Lymphoma	1
		Myoepithelial carcinoma	1
Total	9		

FNAB, fine needle aspiration biopsy; MALT, mucosa-associated lymphoid tissue.

For detection of malignancy, the diagnostic accuracy, specificity and specificity for FNAB were 86.52%, 68.96% and 89.63%, respectively. The Positive Predictive Value (PPV) was 54.05% and the Negative Predictive Value (NPV) was 94.23%. There was moderate agreement between FNAB diagnosis and final histopathological diagnosis (*κ* = 0.52).

In tumors less than 2 cm the sensitivity was 54.54% while in larger tumors it was 77.77%. Also in tumors extending to the deep lobe, sensitivity was 80%. Agreement between FNAB and final histopathological diagnosis was correlated with tumor size (*p* = 0.0) and deep lobe (*p* = 0.004) involvement. The efficacy of FNAB according to deep lobe involvement and tumor size is seen in Table 5, Table 6.

**Table 5:** FNAB efficacy according to deep lobe extension.

	Extension (*n* = 28)	No extension (*n* = 166)
Sensitivity (%)	80	63.15
Specificity (%)	82.35	90.34
PPV (%)	72.72	46.15
NPV (%)	87.5	94.92
Accuracy (%)	81.48	87.19

FNAB, fine needle aspiration biopsy; PPV, positive predictive value; NPV, negative predictive value.

**Table 6:** FNAB efficacy according to tumor size.

	0–2 cm (*n* = 57)	2.1–4 cm (*n* = 119)	>4.1 cm (*n* = 18)
Sensitivity (%)	54.54	75	100
Specificity (%)	82.60	92	93.33
PPV (%)	42.85	60	66.66
NPV (%)	88.37	95.98	100
Accuracy (%)	77.19	89.65	94.11

FNAB, fine needle aspiration biopsy; PPV, positive predictive value; NPV, negative predictive value.

## Discussion

Parotid gland tumors constitute 3% of head and neck tumors.^1^ Benign tumors are more frequent than malignant tumors. In our study, benign tumors were more common with 85.05%, with the most common type being pleomorphic adenoma (43.3%) in accordance with the literature.^3^, ^4^ The second most common benign tumor was Warthin's tumor (23.71%).^3^, ^4^ Malignant tumors were seen in 14.95% of cases with the most common pathologic type being mucoepidermoid carcinoma (4.13%), also in accordance with the literature.^3^ The distribution of benign and malignant final histopathological diagnoses of tumors in our study is shown in Table 7, Table 8, respectively.

**Table 7:** Benign parotid tumors according to final histopathological diagnosis.

Final histopathological diagnosis	Patients	%
Pleomorphic adenoma	84	43.3
Warthin's tumor	46	23.71
Benign epithelial cyst	9	4.65
Lymphoid hyperplasia	5	2.58
Chronic sialadenitis	5	2.58
Chronic granulomatous inflammation	4	2.06
Lipoma	4	2.06
Pilomatrixoma	2	1.03
Basal cell adenoma	2	1.03
Oncocytoma	2	1.03
Monomorphic adenoma	1	0.51
Myoepithelioma	1	0.51
Total	165	85.05

**Table 8:** Malignant parotid tumors according to final histopathological diagnosis.

Final histopathological diagnosis	Patients	%
Mucoepidermoid carcinoma	8	4.13
Acinic cell carcinoma	4	2.07
Diffuse B cell lymphoma	4	2.07
Squamous cell carcinoma	4	2.07
Adenoid cystic carcinoma	3	1.55
Adenocarcinoma	1	0.51
Tubular carcinoma	1	0.51
Ductal carcinoma	1	0.51
Basal cell carcinoma	1	0.51
MALT lymphoma	1	0.51
Myoepithelial cell carcinoma	1	0.51
Total	29	14.95

MALT, mucosa-associated lymphoid tissue.

Parotid gland tumors are more common in males.^3^ In our study, the age of the patients was 47.5 ± 15.88 (7–82). 88 (45.36%) of patients were female and 106 (54.64%) were male. There was slight male predominance in our cases. Benign parotid tumors are most commonly seen in the 5th decade and malignant lesions in the 6th decade.^3^, ^4^ The mean age was 47.2 for benign and 50.2 for malignant tumors. However, there was no significant difference between the mean age of benign and malignant cases in our study (*p* > 0.05).

Clinical examination, imaging and FNAB can be used in preoperative evaluation of parotid gland tumors. High resolution Ultrasound (USG) is the most accepted imaging modality.^5^, ^6^, ^7^ Other imaging techniques are Computed Tomography (CT) and Magnetic Resonance Imaging (MRI). CT and MRI are more expensive and contrast material is needed. Easier implementation and the possibility to perform FNAB with USG guidance are the reasons for choosing USG over CT or MRI.^8^, ^9^ Similarly, our patients underwent USG for imaging and simultaneous FNAB. Brennan et al.^10^ suggested that USG provides adequate information for initial imaging in superficial lobe parotid gland tumors and some difficulties may be encountered in cases extending to the deep lobe. In that case, the use of MRI is preferred. If extension to the deep lobe and/or suspicion of malignancy is detected with either USG or FNAB, CT scan and/or MRI was performed for further evaluation.

Primary treatment of malignant parotid gland tumors is usually surgery. The extent of surgery depends on the histopathologic type.^3^ With correct preoperative diagnosis a better assessment of the possible extent surgery could help the surgeon with preoperative planning and patient counseling since neck dissection and sacrification of the facial nerve may be necessary in case of a malignant tumor. Although imaging techniques provide a lot of information in the evaluation of parotid gland tumors, histopathological or cytological examination should be needed for correct surgical planning. USG-guided tru-cut biopsy or open parotid gland biopsy are not preferred due to risks of serious complications such as deterioration of the tumor capsule and possibility of tumor spread.^5^, ^11^ In 1987, Layfield et al.^12^ conducted a study in which they showed 58% of consistency between FNAB and final histopathologic diagnosis for head and neck tumors. Although the FNAB was described long time ago, it began to gain popularity after this study and nowadays is routinely performed. FNAB is a cheap, fast and easy method for preoperative diagnosis and has a low complication rate and morbidity.^13^, ^14^, ^15^ Rarely, complications such as bleeding, facial nerve injury, fibrosis, and tumor erosion have been reported in the literature.^7^ No complications due to FNAB were observed in our study.

FNAB has been shown to be an important modality in the evaluation of the thyroid gland and lymph node pathologies, but there is no consensus about its use in major salivary gland tumors. The heterogeneous structure of salivary glands has been shown as a reason for a wide range of sensitivity reported in many studies.^16^ According to some researchers, parotid tumors other than pleomorphic adenomas are uncommon and cytopathologists may misdiagnose FNAB if they are not specialized in parotid tumors. Therefore, they suggested that FNAB may be helpful in preoperative planning, but it should not overcome the surgeon's clinical experience and intraoperative findings.^17^

For higher sensitivity, FNAB should be made by experienced clinician. FNAB must include the cortex of parotid gland tumor and samples must be examined the by expert cytopathologists.^11^ Especially in cystic lesions, if the specimen taken from the core and does not contain the cortex, the probability of containing necrotic material increases, leading to non-diagnostic or false negative results. Viguer et al.^18^ recommended aspiration from several points in the same tumor to reduce the false negative result rate. In our study, FNABs were performed by experienced radiologists under USG guidance; syringe was moved in 4–5 directions through the tumor without exiting to obtain enough material for diagnosis and evaluated by expert pathologists.

Reported FNAB sensitivity and specificity values also vary in different populations.^19^, ^20^ Sensitivity varies between 38%^21^ and 97%^22^ and specificity varies between 81%^13^ and 100%.^23^ We have found diagnostic accuracy; sensitivity and specificity were 86.52%, 68.96% and 89.63% for FNAB in detecting malignancy, respectively. The positive predictive value was 54.05% and the negative predictive value was 94.23%.

Tumor size and deep lobe involvement were found to be associated with the efficacy of FNAB.^5^ Ghantous et al. found the accuracy of FNAB to be higher^5^ in patients with parotid gland tumors larger than 24 mm on CT. Sensitivity of FNAB in tumors smaller than 2 cm was 54.54%, and that of larger than 2 cm was 77.77% in our study. In parotid gland tumors with deep lobe extension, FNAB sensitivity was calculated as 80% and in superficial lobe tumors as 63.15%. In our patients, no isolated deep lobe parotid tumor was found; usually cases have a deep lobe extension of the superficial lobe. We think that the higher sensitivity of FNAB in parotid tumors with deep lobe extension may be due to larger size and more patients with isolated deep lobe involvement should be examined.

Our study has some disadvantages due to its retrospective nature. Since pathologists who have studied FNAB specimens for 10 years may have been different, some variability in the interpretation may have occurred. Pathology results which were not available could also have affected our results. However, due to the lower incidence of parotid gland tumors a longer period of time is necessary to accumulate a larger number of cases and these effects can be considered inevitable. Moreover, our results are similar to previously published results.

## Conclusion

FNAB is an important diagnostic tool in the evaluation of parotid gland tumors. The FNAB specificity, sensitivity and accuracy change depending on the location of the superficial and deep lobes of the parotid gland and the size of the tumor. Its accuracy is better for benign tumors. Sensitivity in tumors larger than 2 cm and with extension to deep lobe is higher. The use of FNAB in combination with clinical and radiological evaluation may help reduce false positive and false negative diagnosis.

## Conflicts of interest

The authors declare no conflicts of interest.
